# Novel Allulose- and Amino Acid-Containing Sugar-Free Electrolyte Beverage Improves Body Water Restoration in *Ad Libitum* Rehydration Following Exercise-Induced Dehydration

**DOI:** 10.3390/nu18213601

**Published:** 2026-10-30

**Authors:** Sajjad Rezaei, Sai Tejaswari Gopalakrishnan, Parker Kooima, Abigail E. Wasserbeck, Isabela E. Kavoura, Oviya Srikanth, Vivian Cervantes, Ryan J. Muro, Michael Lelko, Floris C. Wardenaar, Gwyneth W. Gordon, Stavros A. Kavouras

**Affiliations:** 1 Hydration Science Lab, College of Health Solutions, Arizona State University, Phoenix, AZ 85004, USA; srezaei8@asu.edu (S.R.); sgopal52@asu.edu (S.T.G.); aewasser@asu.edu (A.E.W.); ikavoura@asu.edu (I.E.K.); osrikanth@umass.edu (O.S.); vcervan8@asu.edu (V.C.); ramuro@asu.edu (R.J.M.); 2 Athleat Field Lab, College of Health Solutions, Arizona State University, Phoenix, AZ 85004, USA; pkooima@asu.edu (P.K.); floris.wardenaar@asu.edu (F.C.W.); 3 Liquid I.V., El Segundo, CA 90245, USA; 4 The Metals, Environmental and Terrestrial Analytical Laboratory (METAL), Arizona State University, Tempe, AZ 85287, USA; gwyneth.gordon@asu.edu

**Keywords:** fluid balance, oral rehydration solution, sports drink, fluid retention

## Abstract

**Objectives:** This study evaluated the rehydration efficacy of a novel sugar-free oral rehydration solution sweetened with allulose and amino acids (SF-ORS), compared to a traditional sugar-sweetened ORS (SS-ORS) and water. **Methods:** Twenty recreationally active adults (nine females) participated in a randomized crossover study involving three rehydration trials after a ~1.8% body mass loss induced by exercise in heat. Participants consumed *ad libitum* for 4 h one of: (a) SS-ORS (glucose/sucrose: 26 g/L, Na^+^: 49 mmol/L, K^+^: 19 mmol/L, osmolality: 223 mmol/kg); (b) SF-ORS (allulose: ~8 g/L, L-glutamine and L-alanine: ~8 g/L, Na^+^: 46 mmol/L, K^+^: 19 mmol/L, osmolality: 234 mmol/kg); or (c) plain water, all labeled with D_2_O (10 g/L) to trace the appearance of ingested fluid in plasma. **Results:** There was no statistically significant difference in total fluid intake between trials (water: 1603 ± 567, SF-ORS: 1826 ± 728, SS-ORS: 1918 ± 713 mL; *p* = 0.087). At 4 h, fluid retention following SF-ORS (46.6 ± 23.1%) and SS-ORS (39.7 ± 24.0%) was higher than water (24.8 ± 29.3%, all *p* < 0.05), and body weight was better restored following SF-ORS (0.83 ± 0.65%) than water (0.16 ± 0.77%, *p* = 0.011). Urine output at 3 h was lower following SF-ORS (753 ± 394 mL) than SS-ORS (976 ± 583 mL, *p* = 0.035). No significant difference in plasma D_2_O enrichment per gram of D_2_O ingested (average over 4 h) was found between SF-ORS (21.0 ± 5.5 ppm/g) and water (22.0 ± 5.0 ppm/g, *p* = 0.30), while SS-ORS (20.2 ± 4.9 ppm/g) showed lower enrichment per g ingested compared to water (*p* = 0.022). SF-ORS resulted in lower free water clearance than water over the rehydration period (*p* < 0.001). **Conclusions:** An allulose-sweetened, amino acid-based electrolyte beverage may be a viable option for post-exercise rehydration when limiting sugar and energy intake is desired.

## 1. Introduction

During exercise in the heat, sweat evaporation is the primary mechanism for dissipating metabolic heat, particularly in dry environments. Without adequate fluid replacement during exercise, sweating can cause substantial body water loss (dehydration), which in turn impairs thermoregulation [1]. Even a 2% body weight loss can impair endurance performance [2], strength [3], cognitive function [4], mood [5], and overall well-being [6]. Ideally, athletes should consume fluids throughout activity to prevent excessive dehydration (>2% body weight loss) [1]. However, fluid intake during exercise is often lower than sweat losses [7,8], and studies suggest that fluid intake during exercise rarely exceeds 2/3 of fluid losses [7,9,10]. This phenomenon has been studied extensively and described as involuntary dehydration [11]. Therefore, efficient post-exercise rehydration is essential.

Rehydration depends on gastric emptying, intestinal absorption, delivery of absorbed water and solutes to the vasculature, and excretion of ingested fluids. The volume and composition of a beverage strongly influence the efficacy of rehydration [12]. Sports drinks are specifically designed to be consumed during exercise. They are typically formulated as isotonic carbohydrate–electrolyte solutions and can balance two major needs during exercise: fluid homeostasis and fuel delivery to working muscles [13]. The ingestion of a sports drink as a post-exercise rehydration strategy can be justified by the action of the intestinal epithelial sodium-glucose co-transporter 1 (SGLT1), which enhances fluid absorption by the co-transport of glucose and sodium into the intestinal epithelial cells, creating an osmotic gradient that draws water into the cells and onward into the serosal compartment [14,15]. In addition, glucose intake triggers the anti-natriuretic effect of insulin, which enhances renal sodium reabsorption [16]. An increase in plasma sodium and carbohydrates raises plasma osmolality, the main physiological trigger for arginine vasopressin release from the posterior pituitary gland [17]. This response enhances water reabsorption in the renal tubules and suppresses diuresis. However, sodium concentration in most sports drinks (20–30 mmol/L) is at the lower end of the typical human sweat sodium range [18] and may be insufficient for optimal rehydration. It has been suggested that sodium intake greater than 40 mmol/L is needed to significantly improve net fluid balance compared to water [19,20,21,22,23].

A beverage with a carbohydrate concentration of 10–12%, which is greater than that of most sports drinks (~6%), could have higher osmolality (667–691 mmol/kg), even with lower sodium content, and result in slower gastric emptying and intestinal absorption [21,24,25]. Oral rehydration solution (ORS) with lower osmolality (≤ 250 mmol/kg), higher sodium concentration (45–90 mmol/L), but lower carbohydrate (~2.5%) [26] was originally developed to treat diarrhea-induced dehydration, but has gained increasing attention for post-exercise rehydration [27,28,29]. Ly et al. (2023) reported that ORS and sports drinks provide comparable fluid retention, both superior to plain water [28], whereas Fan et al. (2020) demonstrated greater post-exercise fluid retention with ORS compared to a sports drink [27]. The hypotonic formula of an ORS, with higher electrolyte content and lower carbohydrate concentration, likely accelerates intestinal water uptake without adversely affecting gastric emptying or luminal osmolality but yet remains to be directly investigated [30,31].

High consumption of sugar-sweetened beverages is a growing health concern [32] and has prompted increased availability and consumption of sugar-free/low-calorie sweetened beverages as substitutes. This is particularly relevant for recreationally active adults who exercise for health or weight management, as post-exercise rehydration can involve large beverage volumes. Low- and no-calorie sweeteners can enhance palatability, which has been linked to increased libitum fluid intake after exercise [33,34]. However, little is known about the role of non-caloric sweeteners on fluid balance [35].

Allulose (D-allulose or D-Psicose) is a rare sugar found in nature, with 70% of the sweetness of sucrose [36] and negligible energy content. It is rapidly absorbed in the small intestine and excreted in the urine [37]. In healthy adults, allulose has been shown to attenuate postprandial blood glucose and insulin elevations [38]. In the absence of glucose, an allulose-sweetened beverage could also avoid glucose-stimulated intestinal chloride secretion, which draws water into the intestinal lumen [39,40].

Amino acids have been investigated as alternative solutes that enhance intestinal sodium and water transport in the absence of carbohydrate-facilitated fluid delivery in sugar-free ORS [29,41,42,43]. Amino acids are absorbed in the proximal small intestine via sodium-coupled, carrier-mediated transport, thereby stimulating epithelial sodium uptake and promoting water absorption [44]. In an exercise-induced dehydration and rehydration protocol, after replacing 125% of rehydration with an amino acid-based sugar-free ORS, net fluid balance was found to be comparable to a glucose-based ORS and superior to a sugar-free ORS [29]. The rehydration efficacy of an amino acid-based beverage likely depends on the selection of amino acids and their concentrations, given differences in sodium stoichiometry, competition for shared transporters, regional variation in transporter density along the small intestine, and saturation kinetics [45,46]. L-glutamine is one of the amino acids shown to act as a co-transport substrate with sodium, thereby enhancing electrolyte and water absorption from the human jejunum [47]. L-alanine and glutamine appeared more stable than glutamine alone, likely by augmenting ion transport activity in intestinal epithelia [48].

To our knowledge, no study has compared the post-exercise rehydration efficacy of a sugar-free ORS containing L-alanine and L-glutamine with a sugar-based ORS. The aim of the present study was to investigate the impact of *ad libitum* ingestion of amino acid-based ORS sweetened with allulose on fluid balance markers following exercise in the heat in recreationally active adults. We hypothesized that both ORSs would increase fluid intake and fluid retention compared with water and explore whether the allulose-sweetened amino acid-based ORS differed from the sugar-sweetened ORS.

## 2. Materials and Methods

### 2.1. Ethical Approval

Ethical approval for the current study was obtained from the institutional review board at Arizona State University (STUDY00019144; approval date: 14 February 2024) for human experimentation, in accordance with the Helsinki Declaration of 1975, as revised in 1983. This trial was registered at https://clinicaltrials.gov/ under NCT06414291 before subject recruitment began (accessed on 26 October 2026). No changes were made to the protocol, outcomes, eligibility criteria, or beverages after recruitment began. All participants provided written informed consent prior to data collection.

### 2.2. Study Design

In this randomized, counterbalanced crossover design study, participants visited the laboratory four times: one screening visit and three experimental trials. Block randomization was used to determine each participant’s trial order, with a 1–4 week space between trials. Trials were identical except for the beverages provided and trial order followed a Latin square.

### 2.3. Participants

A priori power analysis was conducted using G*Power (version 3.1, Heinrich-Heine-Universität Düsseldorf, Düsseldorf, Germany), based on the difference in net fluid balance between a carbohydrate–electrolyte beverage and a water sweetened with a non-nutritive sweetener, as reported by Wemple et al. (1997) [49]. A sample of 18 participants was required to detect a significant difference between conditions with 80% power and an alpha of 0.05. To account for potential attrition, two additional participants were recruited.

### 2.4. Screening Visit

During the screening visit, body weight and height were measured using an ultrasonic stadiometer (Seca 286 DP; Seca Ltd., Hamburg, Germany). Volunteers were eligible if they were 18–60 years old, trained at least twice per week, had a stable body weight (less than 2.5 kg fluctuation) for >2 months and had a body mass index < 35 kg/m^2^. Exclusion criteria for participation included night shift working, cardiovascular, renal, or hepatic diseases, thyroid medication use, and a bariatric surgery history. Recruitment was conducted via word of mouth, flyers, social media, and online advertisements. Twenty recreationally active healthy adults (11 males and 9 females; age, 26 ± 5 yr; height, 1.69 ± 0.10 m; body mass, 72 ± 11 kg; BMI, 24.9 ± 2.3 kg/m^2^) completed this study. Male participants were aged 25 ± 5 yr with a height of 1.76 ± 0.06 m, body weight of 79 ± 7 kg, and BMI of 25.1 ± 2.0 kg/m^2^. Female participants were aged 28 ± 3 yr with a height of 1.61 ± 0.07 m, body mass of 64 ± 8 kg, and BMI of 24.6 ± 2.7 kg/m^2^. All participants received financial compensation.

### 2.5. Pre-Trial Standardization

Female participants were tested during the early follicular phase of their menstrual cycle to control for hormonal influences on fluid balance. All participants were instructed to avoid exercise training and alcohol intake the day prior to each trial. A standardized meal was provided for the evening before each trial, and participants were instructed to consume an adequate amount of fluid to ensure optimal hydration the following morning. Participants completed a 24 h food and fluid diary before the first trial and were asked to replicate this pattern before subsequent trials. On the morning of each visit, they drank 500 mL of plain water, finishing no later than 30 min before arriving at the laboratory. Participants provided an initial urine sample to verify hydration status (urine specific gravity ≤ 1.020). All experimental trials commenced at the same time of day (07:00–7:20).

### 2.6. Experimental Protocol

To observe the effects of different beverages on rehydration, participants completed a 2 h exercise protocol inducing −1.8% body weight loss (−1.84 ± 0.47%), followed by a 4 h rehydration period consisting of a 500 mL standardized initial bolus followed by *ad libitum intake*. After passing the urine hydration test, participants were provided with a standardized breakfast consisting of a bagel with two tablespoons of cream cheese. A licensed phlebotomist inserted an intravenous catheter into an antecubital vein for subsequent blood sampling. After 20 min of rest, a baseline blood sample was collected at room temperature, the participants emptied their bladders, and a baseline euhydrated body weight was recorded.

### 2.7. Dehydration Phase

After baseline sampling, dehydration up to ~1.8% body weight was induced by exercise at 70% of age-predicted maximum heart rate in a warm environment (31 ± 1 °C, 32 ± 4% relative humidity). Participants completed four 25 min exercise bouts, separated by 5 min rest intervals, alternating between cycling on an ergometer and walking on a treadmill, until subjects reached either a 1.8% body weight loss or a total of 2 h of exercise. Exercise intensity was recorded during the first trial and was repeated for the remaining trials.

### 2.8. Rehydration Phase

After dehydration, participants voided their bladders and rested for 30 min in a temperate environment (21 °C), and a baseline, dehydrated blood sample was collected before beginning the 4 h rehydration period. During the first 5 min of rehydration, subjects consumed a 500 mL standardized initial bolus of the assigned beverage, followed by *ad libitum* intake for the remainder of the period. The beverages were: (a) a sugar-sweetened oral rehydration solution (SS-ORS; Liquid IV Hydration Multiplier^®^, El Segundo, CA, USA); (b) a sugar-free oral rehydration solution (SF-ORS; Liquid IV Hydration Multiplier^®^ Sugar-Free, El Segundo, CA, USA); or (c) plain water (W; AquaFina^®^, PepsiCo, New York, NY, USA). Hydration Multiplier^®^ was selected as a representative commercial ORS [50], while Hydration Multiplier^®^ Sugar-Free contained comparable sodium, potassium, and osmolality, but was sweetened with allulose (~8 g/L) and fortified with L-glutamine and L-alanine (~8 g/L combined). The composition of each beverage is reported in Table 1, and the total amount of each ingredient consumed during each trial is reported in Table S1. All beverages were prepared fresh and enriched with 10 g/L of deuterated water (deuterium oxide 99.9 atom % D, Cambridge Isotope Laboratories, Inc., Tewksbury MA, USA) and served at room temperature in opaque bottles. D_2_O was added as a tracer of the ingested fluid, and the 500 mL bolus therefore contained 5 g of D_2_O. Participants remained blind to the ORS beverage type until all trials were completed.

### 2.9. Experimental Measurements

Fluid intake was recorded at 30, 60, 90, 120, 180, and 240 min of rehydration by weighing the bottles before and after each interval on a calibrated digital scale (FLB FORELIBRA, Nanjing, China), assuming 1 g = 1 mL. Body weight was measured at euhydrated and dehydrated baselines, and at 30, 60, 90, 120, 180, and 240 min of rehydration. Blood samples were collected at euhydrated and dehydrated baselines and 15 time points over the rehydration period to measure D_2_O enrichment. Euhydrated and dehydrated baselines and 30, 60, 120, 180, and 240 min blood samples were analyzed to measure plasma osmolality, total protein, glucose, electrolytes, and copeptin. All blood samples were collected after >20 min of seated rest, without stasis, under temperate environmental conditions (21 °C), prior to subjects using the bathroom. Six urine samples were collected at euhydrated and dehydrated baselines, and at 60, 120, 180, and 240 min of rehydration by weighing the urine containers on the digital scale, assuming 1 g = 1 mL. Perceptual ratings of thirst, stomach fullness, and taste were assessed using a visual analog scale (VAS) before each urine sample.

### 2.10. Sample Analysis

Blood samples were analyzed in duplicate for osmolality, D_2_O enrichment, total protein, glucose, sodium, potassium, and chloride. Blood samples were analyzed immediately in triplicate for hematocrit by the microhematocrit method and hemoglobin by the cyanmethemoglobin method (Drabkin reagent; Sigma-Aldrich, St Louis, MO, USA), and plasma volume changes relative to the dehydrated baseline (0 min) were calculated based on hematocrit and hemoglobin changes using the Dill–Costill equation [51]. Freezing-point depression (A2O Advanced Automated Osmometer, Advanced Instruments Inc., Norwood, MA, USA) was used to measure plasma osmolality. Blood glucose was measured using the HemoCue^®^ Glucose 201 System (HemoCue AB, Ängelholm, Sweden). Plasma, sodium, potassium, and chloride were analyzed using a clinical chemistry analyzer (Beckman Coulter AU 480, Brea, CA, USA). Plasma copeptin was measured with an ultrasensitive sandwich immunoassay (BRAHMS Copeptin proAVP, Kryptor Compact Plus, Thermo Fisher, Hennigsdorf, Germany).

Plasma D_2_O enrichment was used to trace the appearance of ingested fluid in plasma. Plasma D_2_O enrichment was measured on a Los Gatos Research DLT-100 water isotope analyzer (Los Gatos Research, Mountain View, CA, USA) equipped with a CTC-LC PAL autosampler. In brief, samples were diluted gravimetrically with laboratory water enriched to –5.5 ‰ δ^2^H to maintain values within the analytical range. Isotopic enrichment was normalized against certified standards (ER1, ER2, ER4) to the Vienna Standard Mean Ocean Water (VSMOW) scale and expressed in ppm using the absolute D:H ratio of VSMOW. The reproducibility of in-house serum standards prepared identically to study samples is within ±7 ppm. Because beverages were consumed *ad libitum* after the standardized bolus, the amount of D_2_O ingested differed between trials and plasma D_2_O enrichment was therefore expressed per gram of D_2_O ingested. Urine aliquots were analyzed for osmolality (A2O Advanced Automated Osmometer, Advanced Instruments Inc., Norwood, MA, USA), specific gravity (PAL-10S, Atago, Tokyo, Japan), sodium, potassium, and chloride (Beckman Coulter AU 480, Brea, CA, USA). The VAS consisted of a 175 mm line with an anchor at 0 mm (“not at all”) and another anchor at 125 mm labeled “extremely” [52].

### 2.11. Calculations

- Cumulative fluid retention at time point N (%) = [(cumulative fluid intake at N − cumulative urine output at N)/cumulative fluid intake at N] × 100, where N is each urine collection time point (60, 120, 180, and 240 min) and cumulative fluid intake includes the 500 mL initial bolus.
- Body weight change (net fluid balance) at time point N (%) = [(body weight at N − dehydrated body weight)/dehydrated body weight] × 100.
- Urine flow rate at time point N (mL/min) = urine volume at N/collection interval (60 min).
- Osmotic clearance at time point N (mL/min) = (urine osmolality at N × urine flow rate at N)/plasma osmolality at N.
- Free water clearance at time point N (mL/min) = urine flow rate at N − osmotic clearance at N.
- Total osmotic clearance (mL) = sum of osmotic clearance × 60 min.
- Total free water clearance (mL) = sum of free water clearance × 60 min.
- D_2_O enrichment per gram ingested at time point N (ppm/g) = increase in plasma D:H from the dehydrated baseline (0 min) at N/D_2_O ingested at N, where D_2_O ingested (g) = cumulative fluid intake at N (L) × 10 g/L.

### 2.12. Statistical Analysis

Descriptive data are presented as mean ± SD with all statistical analyses being completed using JMP 19 (SAS Institute Inc., Cary, NC, USA). The primary outcomes were total fluid intake, net fluid balance (fluid retention and body weight change), cumulative urine output, and plasma D_2_O appearance. The secondary outcomes were plasma copeptin, plasma volume change, free water and osmotic clearance, thirst, stomach fullness, and taste, and all other variables were exploratory.

Room temperature, relative humidity, baseline values, body weight loss, total osmotic and free water clearance, total fluid intake at 4 h, and fluid retention, body weight change, and urine output at 3 h and 4 h were analyzed using a linear mixed model with one-way repeated measures. Beverage type, trial order, and visits sequence were entered as a fixed effect and participant as a random effect. All other dependent variables, including the time course of the fluid balance outcomes, were analyzed using a linear mixed model with a two-way repeated-measures design, with beverage type, time, their interaction, trial order, and visits sequence were as fixed effects and participant as a random effect. Repeated measurements within each trial were modeled with a first-order autoregressive covariance structure based on the time between samples. This structure fitted better than compound symmetry (lower corrected Akaike information criterion) for all outcomes except plasma osmolality, for which compound symmetry was used. Models were fitted by restricted maximum likelihood, with Kenward–Roger degrees of freedom.

Trial order and treatment sequence were included in all models to account for period and sequence effects, and pre-trial (euhydrated) baselines were evaluated across visits. The model assumptions were checked using residual-by-predicted and normal quantile plots; copeptin, osmotic clearance, urine potassium, and urine osmotic excretion were right-skewed and were log-transformed before analysis. Missing values were not imputed, and the mixed models used all available observations.

Following a significant main effect, pairwise comparisons of estimated marginal means were performed with Tukey’s HSD correction, and comparisons at individual time points were made only when the beverage × time interaction was significant. Differences between beverages are reported with 95% confidence intervals (CI), and Hedges’ g is reported as an effect size for the primary outcomes (Table S2). Statistical significance was set at *p* < 0.05.

## 3. Results

### 3.1. Standardization Procedures

All participants started exercise with the USG below 1.020, and no differences were observed between trials (SS-ORS: 1.008 ± 0.006, SF-ORS: 1.008 ± 0.007, water: 1.008 ± 0.007; *p* = 0.8). Baseline body weights were 71.5 ± 10.5, 71.2 ± 10.6, and 71.6 ± 10.5 kg for SS-ORS, SF-ORS, and water, respectively (*p* = 0.27). Room temperature (31.1 ± 1.1 C, *p* = 0.10) and relative humidity (32 ± 7%, *p* = 0.20) during the dehydration phase did not differ between the trials. Body weight loss during the dehydration was 1.31 ± 0.42 kg (1.82 ± 0.48%), 1.39 ± 0.34 kg (1.94 ± 0.37%), and 1.26 ± 0.44 kg (1.75 ± 0.54%) for SS-ORS, SF-ORS, and water, respectively (*p* = 0.20). Pre-trial (euhydrated) urine specific gravity, body weight, plasma osmolality, plasma sodium, and copeptin did not differ between the beverages (all *p* ≥ 0.25) or across visits (all *p* ≥ 0.28). There was no effect on trial order or visit sequence on statistical models (all *p* > 0.20).

### 3.2. Ad Libitum Fluid Intake

**Figure 1:**
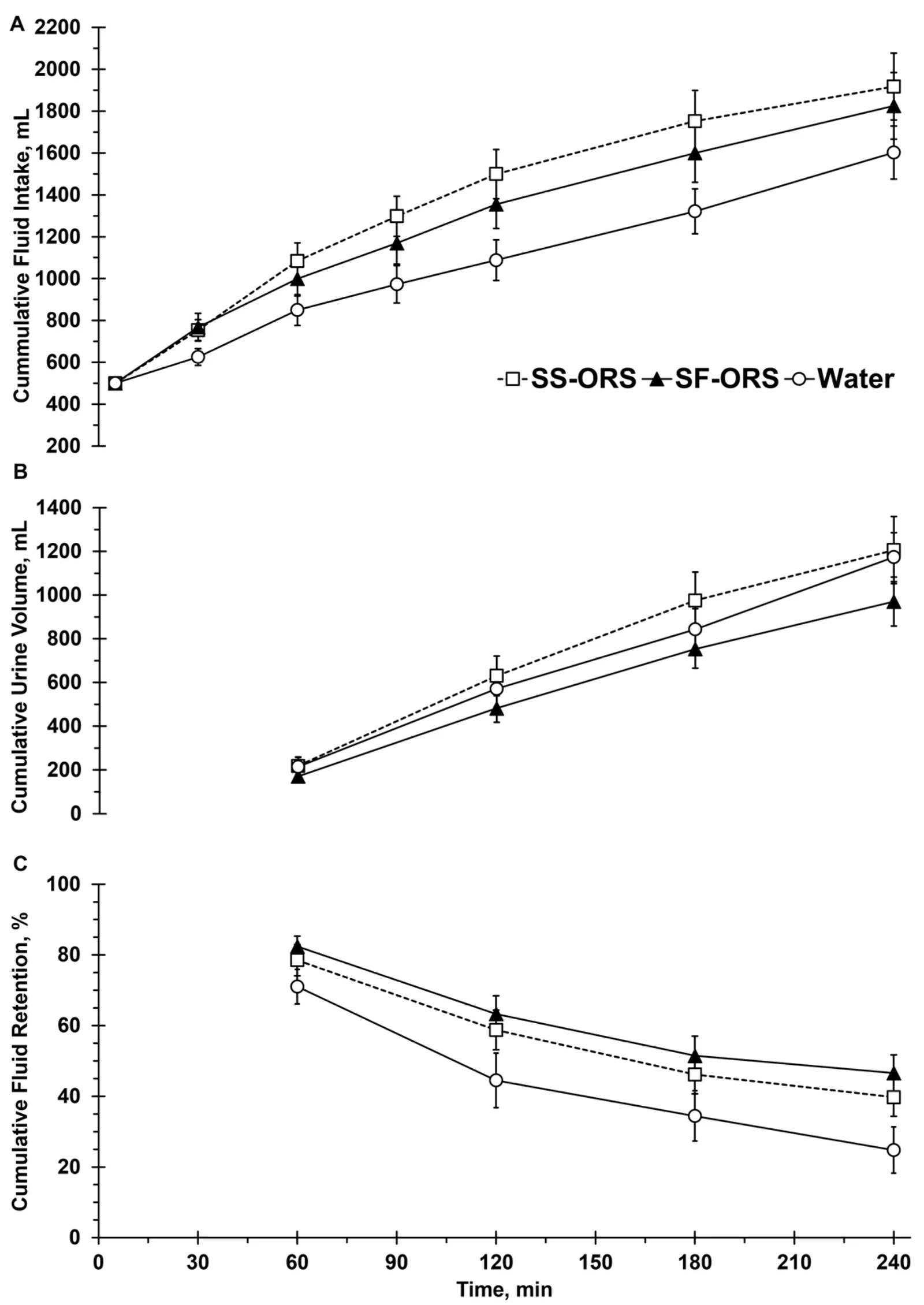
Cumulative fluid intake (**A**), cumulative urine volume (**B**), and cumulative fluid retention (**C**) during the 4 h rehydration period with either a sugar-sweetened oral rehydration solution (SS-ORS), sugar-free oral rehydration solution (SF-ORS), or water. Data are presented as mean ± SE (*n* = 20; 9 females).

Total fluid intake at 240 min was 1603 ± 567 mL, 1826 ± 728 mL, and 1918 ± 713 mL for water, SF-ORS, and SS-ORS, respectively (including the 500 mL standardized bolus; Figure 1A). Voluntary fluid intake after the standardized bolus was 1103 ± 567 mL, 1326 ± 728 mL, and 1418 ± 713 mL for water, SF-ORS, and SS-ORS, respectively. There was no significant main effect of beverages on total fluid intake between beverages (cumulative fluid intake at 240 min, *p* = 0.087). Over the rehydration period (average over 4 h), SS-ORS resulted in greater fluid intake compared to water (45mL [95% CI 1, 90], *p* = 0.048). Across all trials, the greatest proportion of total fluid intake was consumed during the first 30 min of rehydration, which included the standardized 500 mL bolus (716 ± 244 mL), and the second largest proportion was consumed between 30 and 60 min (258 ± 213 mL).

### 3.3. Urine Output and Fluid Retention

There was a main effect of beverage on cumulative urine volume at 180 min (*p* = 0.044), and the SS-ORS resulted higher cumulative urine volume compared to the SF-ORS (−223 mL [95% CI −433, −12], *p* = 0.035). At 240 min, there was a main effect of beverage on total urine output (*p* = 0.049), and there was a trend for lower total urine output following the SF-ORS compared to the SS-ORS (235 mL [95% CI −7, 479], *p* = 0.059).

There was a main effect of beverage on cumulative fluid retention at 180 min (*p* = 0.021), and the SF-ORS resulted in greater cumulative fluid retention compared to water (17% [95% CI 2, 31], *p* = 0.018). At 240 min, there was a main effect of beverage on cumulative fluid retention (24.8 ± 29.3%, 46.6 ± 23.1%, and 39.7 ± 24.0% for water, SF-ORS, and SS-ORS, respectively, *p* = 0.003), and compared to water, cumulative fluid retention was greater following SF-ORS (21% [95% CI 7, 36], *p* = 0.002) and SS-ORS (14% [95% CI 1, 29], *p* = 0.046).

### 3.4. Net Fluid Balance

**Figure 2:**
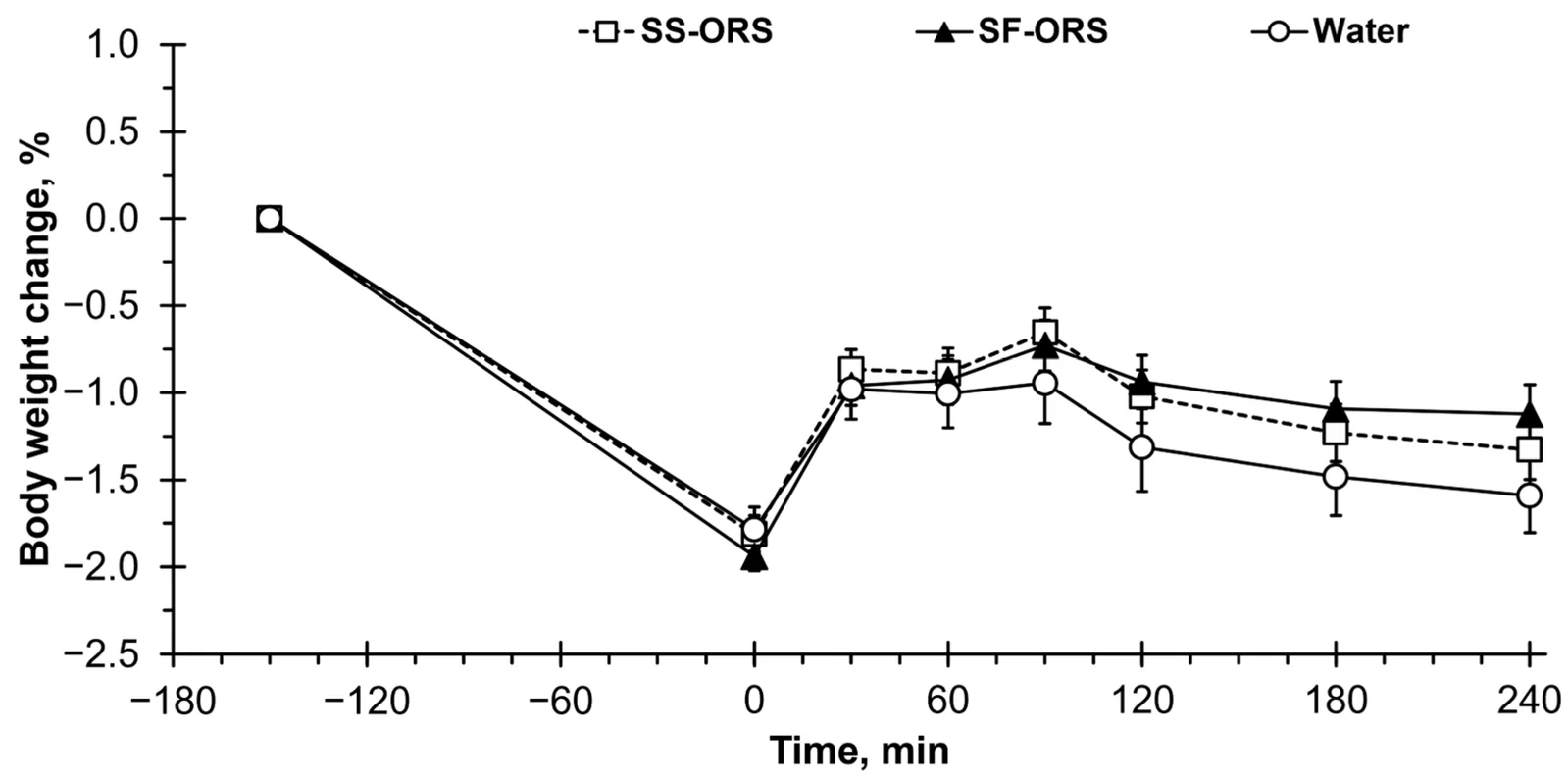
Body weight change from baseline (before exercise, −150 min) and after dehydration (0 min), and over the 4 h rehydration period with either a sugar-sweetened oral rehydration solution (SS-ORS), sugar-free oral rehydration solution (SF-ORS), or water. Data are presented as mean ± SE (*n* = 20; 9 females).

There was a main effect of beverage type on body weight change (from the dehydrated baseline) at 180 min (*p* = 0.015) and 240 min (*p* = 0.011; Figure 2). At 180 min, body weight was more restored following the SF-ORS than water (0.59% [95% CI 0.11, 1.06], *p* = 0.011). At 240 min, body weight change from the dehydrated baseline was 0.16 ± 0.77%, 0.83 ± 0.65%, and 0.49 ± 0.58% for water, SF-ORS, and SS-ORS, respectively, and SF-ORS resulted in greater body weight restoration than water (0.67% [95% CI 0.15, 1.19], *p* = 0.011). Over the rehydration period (average over 4 h), there was a main effect of beverage on body weight change (*p* = 0.021), and SF-ORS showed significantly greater body weight restoration than water (0.39% [95% CI 0.06, 0.72], *p* = 0.017). All participants finished the rehydration period in a negative net fluid balance across all trials.

### 3.5. Plasma D_2_O Enrichment, Volume Change, and Osmolality

Table 2 shows D_2_O ingested and enrichment per gram of D_2_O ingested over 4 h rehydration. D2O ingested (average over rehydration) in the SS-ORS was higher than in water (3.0 g [95% CI 0.2, 5.9], *p* = 0.033). There was a main effect of beverage for D_2_O enrichment per gram ingested (*p* = 0.029), and the SS-ORS resulted in lower enrichment than water (−1.7 ppm/g [95% CI −3.2, −0.2], *p* = 0.022). There was no significant difference between SF-ORS and water or between SF-ORS and SS-ORS (all *p* ≥ 0.30). Absolute plasma D_2_O enrichment data are presented in Figure S1.

**Figure 3:**
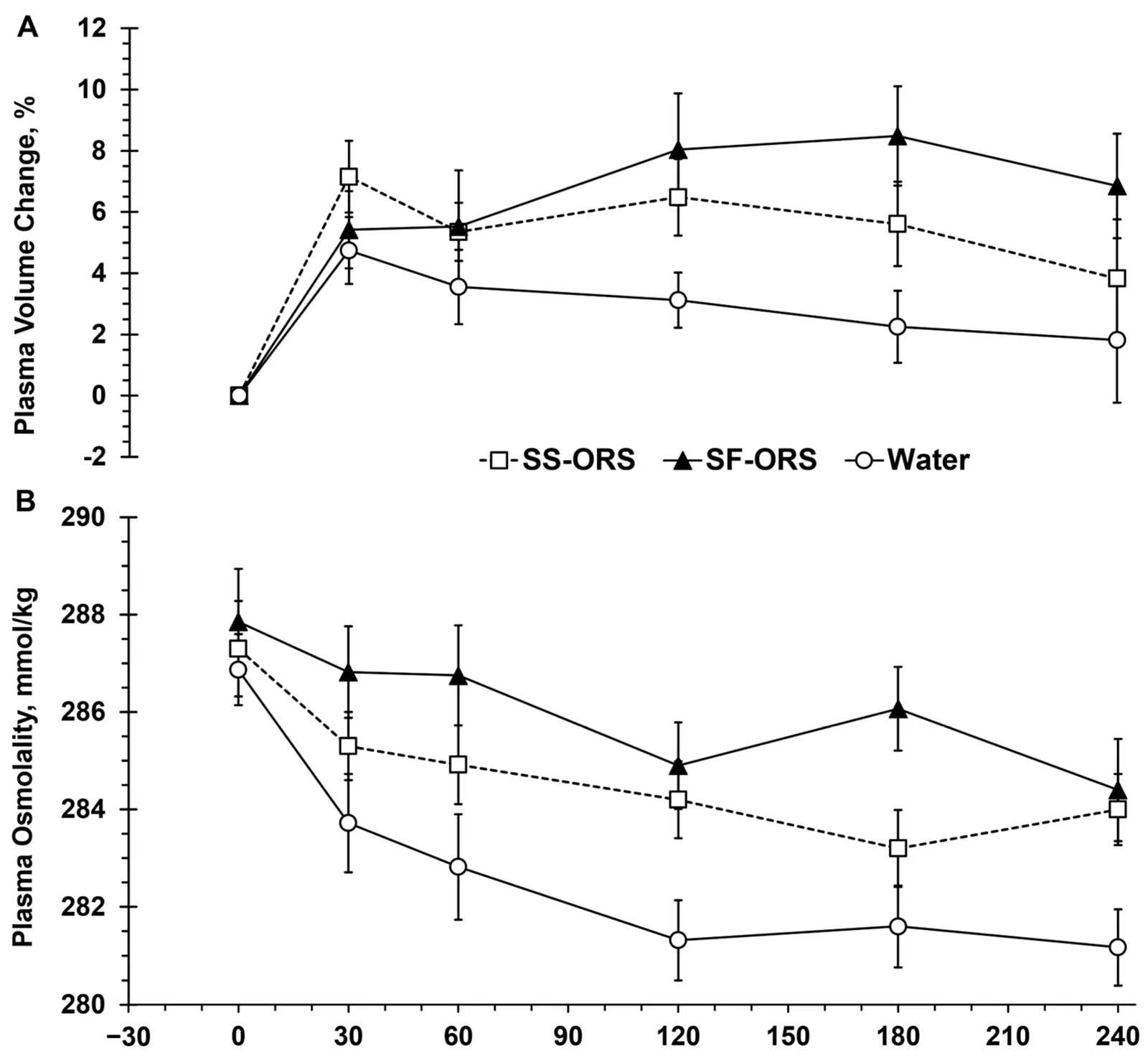
Plasma volume change (**A**), and plasma osmolality (**B**) over the 4 h rehydration with either a sugar-sweetened oral rehydration solution (SS-ORS), sugar-free oral rehydration solution (SF-ORS), or water. Data are presented as mean ± SE (*n* = 20; 9 females).

Figure 3A shows plasma volume change during the rehydration period. There was a main effect of beverage on plasma volume change over 4 h rehydration (*p* = 0.043), and the SF-ORS yielded higher average plasma volume expansion (+3.1% [95% CI 0.2, 5.9], *p* = 0.039).

Plasma osmolality increased during the dehydration phase and then decreased over the rehydration period across all trials (time, *p* < 0.001; Figure 3B). Over 4 h rehydration, plasma osmolality following water was lower than that of the SF-ORS (−3.1 mmol/kg [95% CI −4.9, −1.4], *p* < 0.001) and lower than that of the SS-ORS (−1.9 mmol/kg [95% CI −3.6, −0.2], *p* = 0.025), with no significant difference between SF-ORS and SS-ORS (*p* = 0.194).

### 3.6. Free Water and Osmotic Clearance

**Figure 4:**
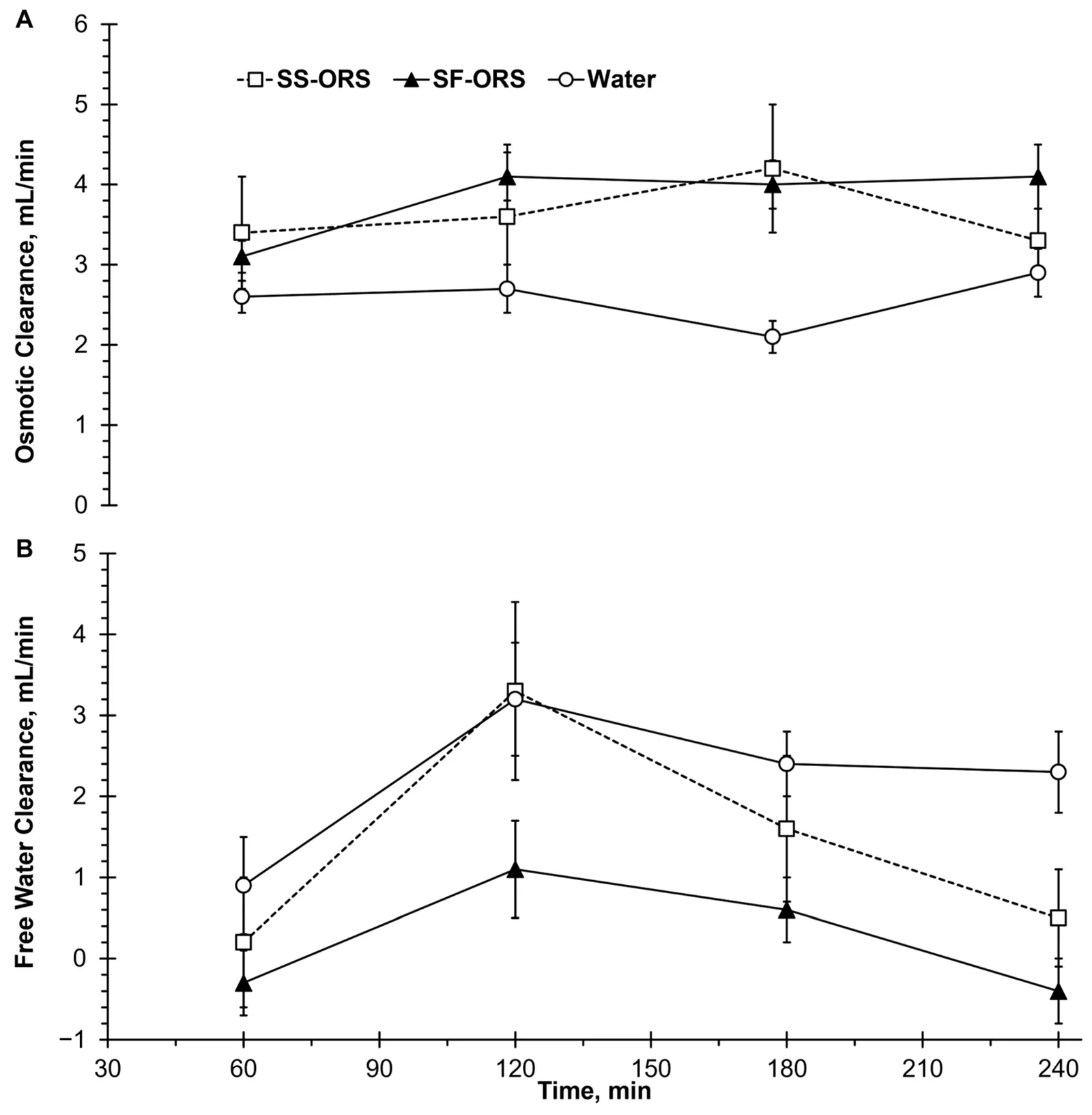
Osmotic clearance (**A**) and free water clearance (**B**) during the 4 h rehydration with either a sugar-sweetened oral rehydration solution (SS-ORS), sugar-free oral rehydration solution (SF-ORS), or water. Data are presented as mean ± SE (*n* = 20; 9 females).

There was a main effect of beverage on osmotic clearance over 4 h rehydration (*p* < 0.001), with an interaction between beverage and time (*p* = 0.014; Figure 4A), and osmotic clearance following water was significantly lower than those of SF-ORS (−1.2 mL/min [95% CI −1.9, −0.5], *p* < 0.001) and SS-ORS (−1.0 mL/min [95% CI −1.7, −0.3], *p* = 0.001), with no significant difference between SF-ORS and SS-ORS trials (*p* = 0.78). There was a main effect of beverage type on free water clearance over the 4 h rehydration period (*p* = 0.001; Figure 4B), and SF-ORS resulted in lower free water clearance than water (−2.0 mL/min [95% CI −3.3, −0.7], *p* < 0.001).

### 3.7. Plasma Copeptin

**Figure 5:**
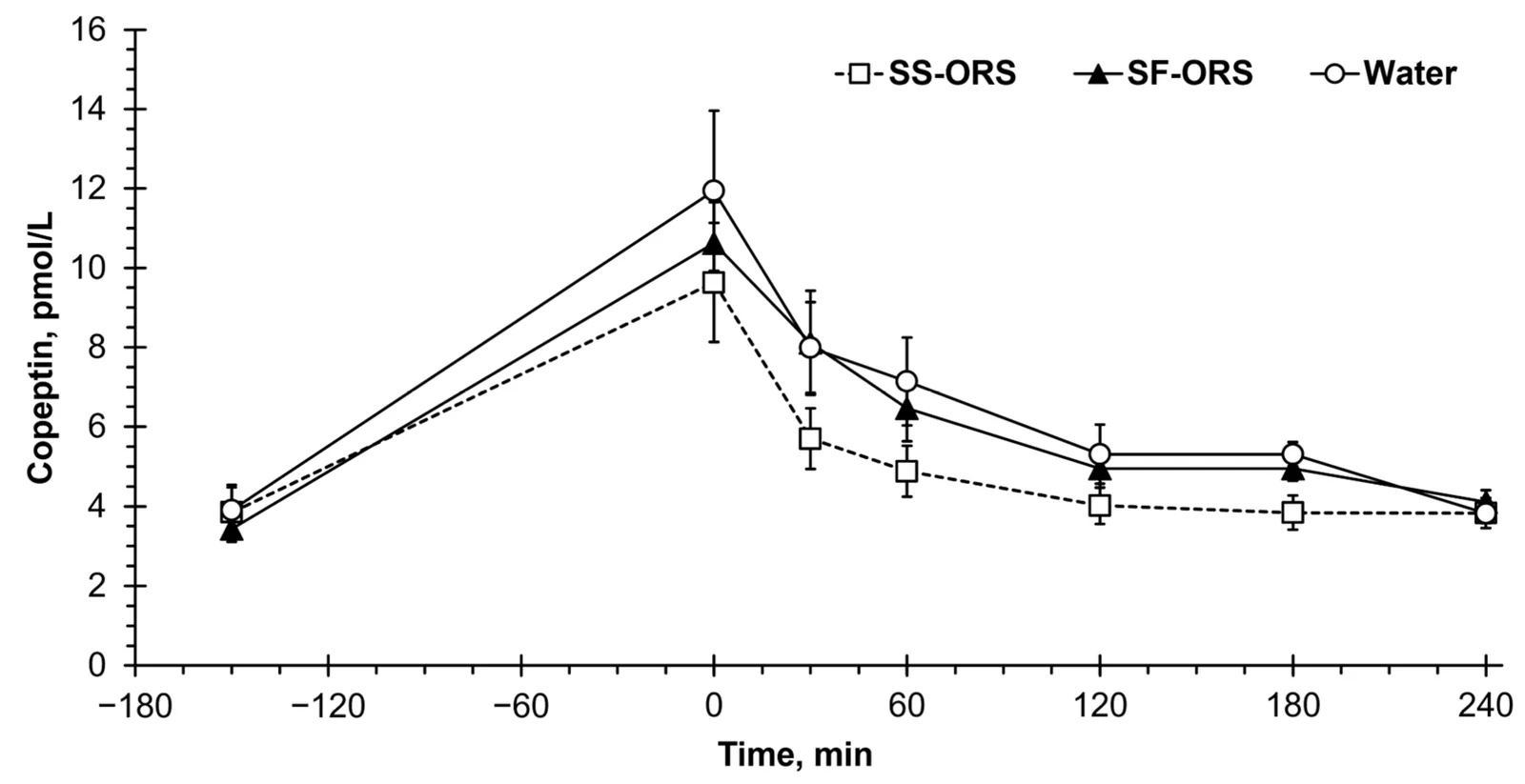
Plasma copeptin concentration before (−150 min) and after dehydration (0 min), and over the 4 h rehydration period with either a sugar-sweetened oral rehydration solution (SS-ORS), sugar-free oral rehydration solution (SF-ORS), or water. Data are presented as mean ± SE (*n* = 20; 9 females).

There was no significant main effect of the beverage type (*p* = 0.15) and no interaction between beverage type and time (*p* = 0.32) for plasma copeptin (Figure 5). There was a main effect of time (*p* = 0.001), and plasma copeptin peaked at the end of dehydration (0 min: 10.7 ± 6.9 pmol/L), higher than other time points (all *p* < 0.01).

### 3.8. Blood Glucose, Electrolytes, and Total Protein

**Figure 6:**
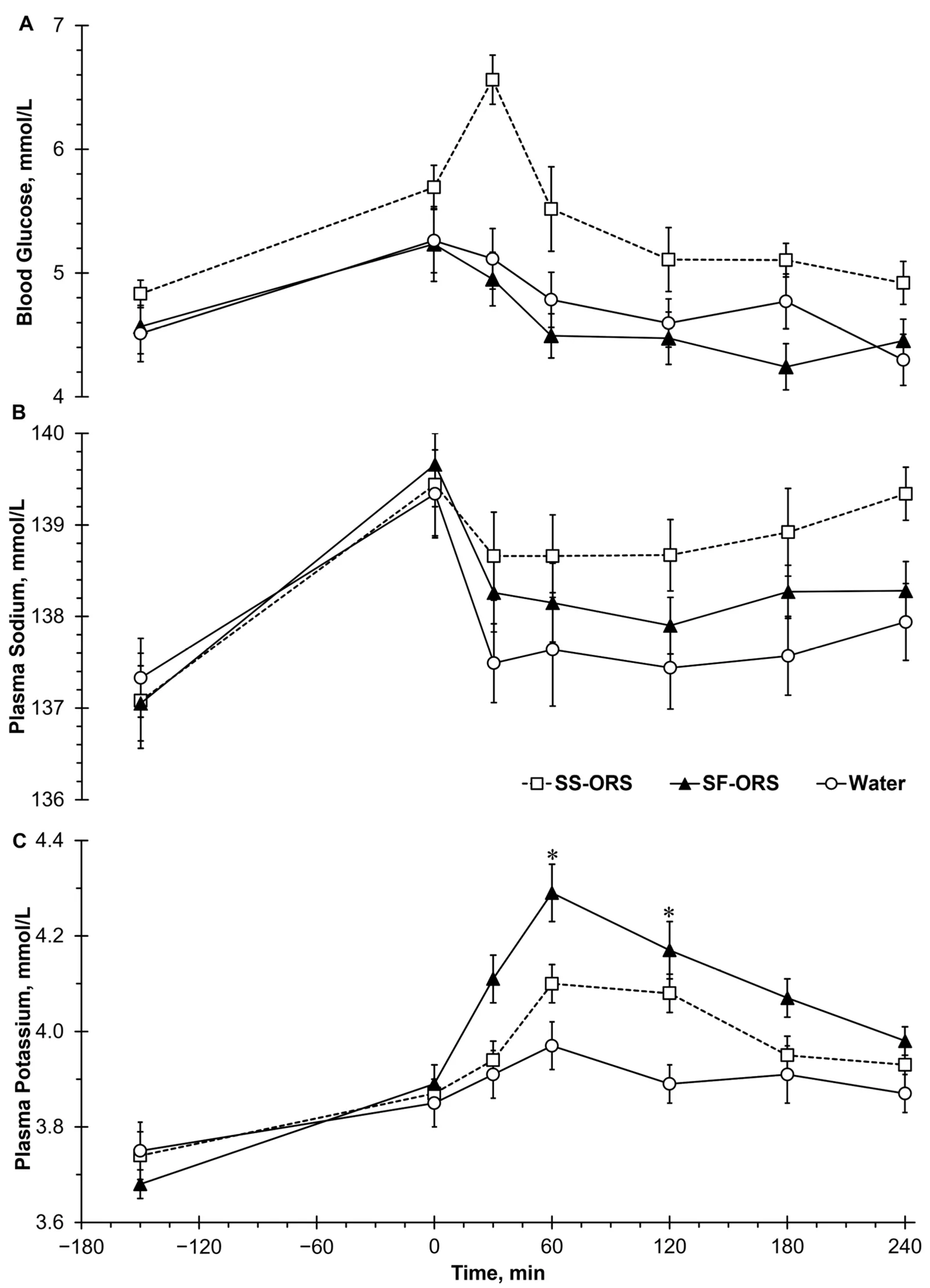
Blood glucose (**A**), plasma sodium (**B**), and plasma potassium (**C**) before (−150 min) and after dehydration (0 min), and over the 4 h rehydration period with either a sugar-sweetened oral rehydration solution (SS-ORS), sugar-free oral rehydration solution (SF-ORS), or water. Data are presented as mean ± SE (*n* = 20; 9 females). Statistical significance was set at *p* < 0.05. * indicates the significant difference compared to water.

The beverage type showed a main effect for blood glucose (*p* < 0.001, Figure 6A). Over 4 h rehydration, blood glucose following the SS-ORS was higher than that of the SF-ORS (0.84 mmol/L [95% CI 0.56, 1.1], *p* = 0.001) and water (0.7 mg/dL [95% CI 0.43, 0.97], *p* < 0.001). There was no significant difference in blood glucose between SF-ORS and water (*p* = 0.46).

Over the study protocol, beverage showed a main effect for plasma sodium (*p* < 0.001; Figure 6B). Over 4 h rehydration, plasma sodium following SS-ORS was higher than that of water (1.1 mmol/L [95% CI 0.4, 1.7], *p* < 0.001). There was no statistically significant difference in plasma sodium between SF-ORS and water (0.5 mmol/L [95% CI −0.2, 1.1], *p* = 0.50). Across all trials, the highest concentration was observed at the end of dehydration (0 min: 139.4 ± 2.2 mmol/L), while the lowest was observed at baseline prior to dehydration protocol (−150 min: 137.1 ± 2.0 mmol/L).

The beverage type showed a main effect for plasma potassium over 4 h rehydration (*p* < 0.001; Figure 6C), and the SF-ORS resulted in significantly greater plasma potassium than water (0.2 mmol/L [95% CI 0.1, 0.3], *p* < 0.001) and SS-ORS (−0.1 mmol/L [95% CI −0.2, −0.1], *p* < 0.015). There was an interaction between time and beverage type (*p* = 0.03), and plasma potassium was significantly higher in SF-ORS compared to water at 60 and 120 min (both *p* < 0.001, Figure 6C).

There was no main effect of beverage for plasma chloride over the rehydration period (*p* = 0.051). Similarly, beverage showed no main effect for total plasma protein over the rehydration period (*p* = 0.164).

### 3.9. Urine Osmolality and Electrolytes

Urine osmolality and electrolytes are presented in Table S3. Over the rehydration period, SF-ORS showed higher osmolality than water (102 mmol/kg [95% CI 17, 186], *p* = 0.014). Over the rehydration period, osmotic excretion following water was lower than that of SF-ORS (−22 mmol [95% CI −34, −10], *p* < 0.001) and SS-ORS (−18 mmol [95% CI −30, −6], *p* = 0.001). The SF-ORS also showed higher osmotic excretion than the SS-ORS (*p* = 0.004). At the end of the rehydration period, the SF-ORS resulted in higher cumulative osmotic excretion than water (87 mmol [95% CI 12, 162], *p* = 0.019).

Over the rehydration period, urine sodium following water was lower than that of the SF-ORS (−24 mmol/L [95% CI −43, −5], *p* < 0.010). There was a main effect of beverage for sodium excretion and water showed lower sodium excretion than that of SF-ORS (−4 mmol [95% CI −6, −2], *p* < 0.001) and SS-ORS (−3 mmol [95% CI −5, −1], *p* = 0.004). At the end of the rehydration period, water resulted in lower cumulative sodium excretion than that of SF-ORS (−15 mmol [95% CI −23, −6], *p* < 0.001) and SS-ORS (−11 mmol [95% CI −19, −3], *p* = 0.004).

Over the rehydration period, urine potassium following water was lower than SF-ORS (*p* = 0.029) and SS-ORS (*p* = 0.003]. Potassium excretion following water was lower than that of SF-ORS (−2.1 mmol [95% CI −3.8, −0.5], *p* = 0.005) and SS-ORS (−2.0 mmol [95% CI −3.6, −0.3], *p* = 0.011). There was an interaction of beverage by time for potassium excretion (*p* = 0.017). At 120 min, water showed lower potassium excretion compared to SF-ORS (*p* = 0.039), and lower than SS-ORS at 180 (*p* = 0.003). At the end of the rehydration period, water resulted in lower cumulative potassium excretion than SF-ORS (8.5 mmol [95% CI 1.9, 15.1], *p* = 0.008) and SS-ORS (8.0 mmol [95% CI 1.3, 14.6], *p* = 0.014).

There was no main effect of beverage for urine chloride over the rehydration period (*p* = 0.118). Beverage showed the main effect for chloride excretion over the rehydration period (*p* = 0.001), and SF-ORS resulted in higher chloride excretion (4.4 mmol [95% CI 1.7, 7.1], *p* < 0.001). At 120, 180, and 240 min, SF-ORS showed higher chloride excretion than water (all *p* < 0.03). At the end of the rehydration period, SF-ORS resulted in higher cumulative chloride excretion than water (17 mmol [95% CI 6, 27], *p* = 0.001).

### 3.10. Perceptual Data

Perceptual ratings are presented in Table S4. There was no main effect of beverage on thirst perception (*p* = 0.105) and stomach fullness (*p* = 0.27). There was a main effect of beverage on taste pleasantness over the rehydration period (*p* = 0.047), and water was rated less palatable than SF-ORS (20 mm [95% CI 1, 41], *p* < 0.049).

## 4. Discussion

This study demonstrated that following exercise-induced dehydration (~1.8% body weight loss) and a 4 h rehydration period consisting of a standardized 500 mL bolus followed by *ad libitum* fluid intake, an amino acid-based ORS sweetened with allulose (SF-ORS) and a sugar-based ORS (SS-ORS) increased fluid retention compared with water at the end of rehydration. Only the SF-ORS improved net fluid balance (body weight restoration), plasma volume expansion compared to water, and lowered free water clearance compared with both water and SS-ORS. Total fluid intake (cumulative fluid intake at 240 min) was 220–320 mL greater with the ORSs than with water, although this difference was not statistically significant. Urine output was lower following the SF-ORS than the SS-ORS at 3 h, but no statistically significant differences were detected between SF-ORS and SS-ORS in total fluid intake, fluid retention, or body weight change. Plasma D_2_O enrichment per gram of D_2_O ingested in SS-ORS was lower than water and did not differ between SF-ORS and water or between SF-ORS and SS-ORS. Both ORSs increased plasma osmolality compared with water, the SS-ORS increased blood glucose and plasma sodium, and the SF-ORS increased plasma potassium compared with water. These results suggest that an amino acid-based ORS sweetened with allulose is a viable option to promote post-exercise rehydration when limiting sugar and energy intake is desired.

The *ad libitum* drinking strategy was chosen to simulate real-life rehydration behavior. In the present study, mean total fluid intake was 132%, 146%, and 127% of the body weight loss following SF-ORS, SS-ORS, and water, respectively, which is within the range recommended as the post-exercise volume intake [53]. Taste pleasantness was rated higher for SF-ORS than water. Flavored, sweetened beverages have been previously shown to promote greater voluntary intake during and after exercise [33,34], and allulose in SF-ORS achieved this palatability benefit without contributing excess sugar intake, supporting its functionality in hydration products.

These superior fluid retention responses to both ORSs, and the greater body weight restoration with SF-ORS are consistent with previous studies comparing carbohydrate–electrolyte solutions containing sodium ≥40 mmol/L with water or beverages containing sodium ≤30 mmol/L [19,20]. Under *ad libitum* drinking, fluid retention reflects both the volume consumed and the renal response. Retention was high early in rehydration with all beverages, because little time had elapsed for urine formation. This difference in fluid retention between the ORS and water widened otastever time (from approximately 9 and 5 percent at 60 min to 21 and 14 percent at 240 min for SF-ORS and SS-ORS, respectively), consistent with the sodium content of the ORS attenuating the diuresis that follows water ingestion. Because participants drank more of the ORS but did not produce more urine than with water, a larger fraction of the ingested fluid was retained. Urine output was lower with the SF-ORS than with the SS-ORS at 3 h, despite similar fluid intake, but the difference was not statistically significant at 4 h, and fluid retention and body weight change did not differ significantly between the ORS beverages. The lower urine output with SF-ORS therefore did not translate into a greater whole-body rehydration (net fluid balance), even though SF-ORS resulted in greater expansion of the intravascular compartment (plasma volume change). The effect size for body weight change between SF-ORS and SS-ORS at 4 h was moderate (Hedges’ g = 0.53; Table S2), and a larger study designed to compare the two ORSs would be needed to determine whether they differ in urine output, fluid retention, and body weight change.

Deuterium oxide dilution is a non-invasive integrated measurement of gastric emptying and intestinal absorption [12]. Jeukendrup et al. (2009) reported that 3% glucose in the beverage led to greater plasma D_2_O enrichment than that of water and higher carbohydrate concentrations, independent of added sodium (20 mmol/L) [54]. The authors speculated that the low glucose concentration did not slow gastric emptying, but facilitated intestinal absorption of sodium and water coupled with glucose through co-transportation via SGLT1 [15]. Under the *ad libitum* drinking we used in the current study, plasma enrichment depends on the amount of tracer ingested, the timing of ingestion, its distribution across body compartments and loss in urine. Although there were no statistically significant differences in total fluid intake (cumulative fluid intake at min 240) between beverages, more D_2_O was ingested over the rehydration period with the SS-ORS than with water, and a fixed volume containing a single tracer dose ingested at a standardized time is required to accurately assess gastric emptying and intestinal absorption [55]. The lower enrichment per gram with the SS-ORS was associated with the greatest amount of D_2_O ingested, suggesting that a larger and more recently ingested tracer dose may not yet have equilibrated with body water, rather than a slower appearance of ingested fluid. Because the amount and timing of each drink differed between participants and trials, these data cannot distinguish differences in gastric emptying or intestinal absorption between beverages, and the D_2_O findings should be interpreted cautiously. Therefore, plasma D_2_O enrichment in the current study is presented as an indicator of the intestinal uptake of the ingested fluid, which results from both the volume and the composition of the beverages consumed, and differences between beverages may reflect differences in voluntary intake driven by taste and other perceptual responses rather than an effect of beverage composition on absorption.

Compensatory fluid regulation is triggered primarily by elevated plasma osmolality and, to a lesser extent, by reduced plasma volume [56]. Post-exercise rehydration with water lowers plasma osmolality, suppresses arginine vasopressin secretion, and increases urine output [56]. However, in our study, there were no significant differences in plasma copeptin, a surrogate marker of arginine vasopressin, between beverages, although plasma osmolality was lower with water than with either ORS. The differences in plasma osmolality between the beverages were small (about 2–3 mmol/kg) and may have been too small to produce detectable differences in copeptin.

Plasma osmolality was higher with both ORS than with water, consistent with the sodium content of the ORS limiting the fall in plasma osmolality during rehydration but did not differ significantly between SF-ORS and SS-ORS. Free water clearance was lower with the SF-ORS than water, indicating that less solute-free water was excreted following the SF-ORS, without a significant difference in plasma osmolality or copeptin between the ORS. The mechanisms underlying this renal response cannot be determined from the present data, and the following explanations remain speculative. Allulose is absorbed in the intestine via glucose transporter 5, independent of sodium [57], and does not generate the electrochemical gradient that drives paracellular water absorption as glucose does via SGLT1. Once absorbed, allulose reaches peak plasma levels within one hour and is not metabolized, resulting in renal excretion of 50–70% of the absorbed dose [57]. Allulose may persist in circulation as an osmotically active solute and may have contributed to sustained plasma osmolality, so circulating allulose could act as an additional osmotically active solute. Similarly, L-glutamine and L-alanine are absorbed via sodium-coupled amino acid transporters such as B^0^AT1 [44], which could support intestinal sodium and water uptake independently of SGLT1. However, plasma and urine allulose concentrations and plasma amino acid concentrations were not measured, and plasma sodium was higher than water only following the SS-ORS. Moreover, the multi-ingredient beverages do not allow the effects of allulose and amino acids to be separated; these hypotheses therefore require testing ingredients to be done separately.

The inclusion of glucose creates a local osmotic gradient across the intestinal epithelium, facilitating the appearance of sodium and fluid in the vasculature via SGLT1, thereby contributing to blood volume expansion [14,15,16]. In the current study, plasma volume peaked at 30 min following the SS-ORS (Figure 3A), paralleling blood glucose responses to the 500 mL standardized bolus (Figure 6A), although plasma volume change over the rehydration period was greater than that of water only following the SF-ORS. Plasma volume is also a function of the volume ingested and the volume excreted by the kidneys, and the greater plasma volume expansion with the SF-ORS may therefore partly reflect its greater fluid retention rather than greater intestinal fluid absorption. On the other hand, the SF-ORS did not raise blood glucose above water, as expected for a beverage containing no glucose. The carbohydrate in the SS-ORS may also explain why only SS-ORS increased plasma sodium and only SF-ORS increased plasma potassium compared with water, as glucose promotes intestinal sodium absorption via SGLT1 [14,15], and the resulting insulin response enhances renal sodium reabsorption [16] and promotes cellular potassium uptake [58]. This explanation remains speculative because insulin was not measured in the current study.

Given that a variety of amino acid-sodium co-transporters exist and glucose may not be ideal in all rehydration contexts, amino acids have been considered as an alternative to glucose in ORS formulation [29,42,43]. Peden et al. reported that an amino acid-based sugar-free ORS resulted in net fluid balance comparable to a glucose-based ORS and greater than a sugar-free ORS [29]. However, the markedly higher sodium content of the amino acid-based sugar-free ORS (76 ± 3 mmol/L) compared to glucose-based ORS (45 ± 0 mmol/L) and sugar-free ORS (31 ± 1 mmol/L) confounded interpretation of the independent contribution of amino acids to rehydration efficacy. Moreover, participants in that study consumed cereal bars providing 1.5 g of carbohydrate per kg body weight during the rehydration period, which may have engaged the SGLT1 pathway and thereby offset the absence of glucose in amino acid-based ORS. Together, our findings and those of Peden et al. warrant further studies isolating the amino acid type from sweetener type at comparable electrolyte concentrations [29].

At the level of dehydration in the current study, the SS-ORS provided ~49 g of sugar, which exceeds the American Heart Association recommendation for daily added sugar intake [59], whereas the SF-ORS provided no sugar and 73 ± 29 kcal. At the end of rehydration, fluid retention following SF-ORS and SS-ORS was 21 and 14 percentage greater than water (Hedges’ g = 0.79 and 0.54, Table S2), and body weight was restored by 0.67% more with the SF-ORS than with water (≈470 g for a 70 kg adult; g = 0.90). The 315 mL greater beverage intake in the SS-ORS, although not statistically different from that of water, corresponds to approximately one quarter of the body weight lost. Whether these differences are practically important depends on the context. It is extremely common and recommended to consume solid food and/or energy-dense beverages in the hours after exercise, which can affect fluid retention when consumed with adequate fluids [60]. Consuming food and/or energy-dense beverages intake may slow gastric emptying and the delivery of fluid and solutes to the circulation, potentially affecting how beverage composition influences net fluid balance and hydration biomarkers [12]. Therefore, these findings are most relevant when food intake is limited or impractical, such as during short recovery windows between competitions or consecutive training sessions, and for recreationally active adults who exercise for health or weight management and wish to limit sugar and energy intake while rehydrating.

It is important to recognize the study’s limitations. *Ad libitum* drinking did not aim to systematically restore body weight loss over a set period, and the time course of fluid intake differs among beverages. The fraction of fluid retained in the gastrointestinal system during later stages of *ad libitum* drinking could not be assessed, complicating comparisons of the primary outcomes at the given time points. The D_2_O tracer was ingested with every drink rather than as a single standardized dose, which limits its use for assessing fluid absorption. In addition, D_2_O ingested was calculated from beverage weight (assuming 1 g = 1 mL) and small differences in beverage density may have resulted in small differences between the assumed and actual D_2_O dose ingested with each beverage. The modest level of dehydration (~1.8% body weight loss) may also limit the generalizability of the findings to larger fluid deficits. Moreover, fluid distribution between extracellular and intracellular compartments was not measured, and the timing of fluid distribution after intestinal absorption remains unclear, as sodium-coupled glutamine uptake may have shifted water into cells rather than expanded plasma volume [61]. Given that enterocytes oxidize glutamine, future work should measure plasma glutamine to distinguish intestinal utilization from systemic availability. In addition, plasma or urine allulose concentrations were not measured, and thus its direct contribution to plasma osmolality and renal water handling cannot be quantified. Almost 30% of ingested allulose escapes small intestinal absorption and reaches the microbiota, potentially drawing water osmotically into the lumen, although human studies have shown minimal fermentation and limited intestinal effects at doses comparable to those used in the present study [57]. In addition, the study was powered to detect differences between a carbohydrate–electrolyte beverage and water, not to establish equivalence between the two ORS beverages; the absence of statistically significant differences between SF-ORS and SS-ORS should therefore not be interpreted as evidence of equivalence. Moreover, total fluid intake varied widely between participants (SD 567–728 mL), and the study, which was powered on net fluid balance, may have been underpowered to detect differences in *ad libitum* intake of the size observed (220–320 mL). Finally, because this study used commercially available products, we cannot isolate the independent contributions of allulose, specific amino acids, or their interaction.

## 5. Conclusions

Following exercise-induced dehydration, both a proprietary blend of allulose and amino acid ORS and a sugar-sweetened ORS improved fluid retention compared with water after a 4 h rehydration period consisting of a standardized bolus followed by *ad libitum* intake, and the sugar-free ORS also restored more body weight than water. No statistically significant differences were detected between the two ORSs in fluid intake, fluid retention, or body weight change. SF-ORS may be a viable option for post-exercise rehydration when limiting sugar and energy intake is desired.

## Figures and Tables

**Table 1:** Composition of the study beverages.

Component	SS-ORS	SF-ORS	Water
Carbohydrate (glucose/sucrose), g/L	26	—	—
Allulose, g/L	—	8	—
L-glutamine + L-alanine, g/L	—	8	—
Sodium, mmol/L (mg/L)	49 (1127)	46 (1058)	—
Potassium, mmol/L (mg/L)	19 (743)	19 (743)	—
Energy, kJ/L (kcal/L)	418 (100)	167 (40)	—
Osmolality, mmol/kg	223	234	—

Values are manufacturer-reported for the sugar-sweetened oral rehydration solution (SS-ORS; Liquid IV Hydration Multiplier^®^) and the sugar-free oral rehydration solution (SF-ORS; Liquid IV Hydration Multiplier^®^ Sugar-Free); water was AquaFina^®^ purified water. L-glutamine + L-alanine is the combined amino acid content (1:1 ratio). D_2_O was added to all beverages as a tracer. —, not present in the beverage.

**Table 2:** Plasma D_2_O enrichment per gram of D_2_O ingested.

Time, min	30	60	90	120	180	240
D_2_O ingested, g						
SS-ORS	7.5 ± 2.2	10.8 ± 3.8	13.0 ± 4.3	15.0 ± 5.2	17.5 ± 6.5	19.2 ± 7.1
SF-ORS	7.7 ± 3.0	9.9 ± 3.7	11.7 ± 4.5	13.6 ± 5.2	15.9 ± 6.3	18.3 ± 7.3
Water	6.3 ± 1.8	8.5 ± 3.3	9.7 ± 4.0	10.9 ± 4.3	13.2 ± 4.8	16.0 ± 5.7
D_2_O enrichment per gram ingested, ppm/g						
SS-ORS	18.3 ± 5.7	18.8 ± 5.3	21.6 ± 4.9	20.6 ± 4.6	20.8 ± 4.1	21.3 ± 3.9
SF-ORS	18.6 ± 6.5	20.5 ± 5.7	21.4 ± 4.8	21.6 ± 5.2	22.1 ± 6.1	21.9 ± 4.2
Water	22.1 ± 7.1	22.4 ± 4.9	22.4 ± 4.6	22.2 ± 4.9	21.2 ± 4.3	21.5 ± 3.8

Data are presented as mean ± SD (*n* = 20; 9 females). D_2_O ingested is the cumulative fluid intake multiplied by the D_2_O concentration of the beverages (10 g/L). Enrichment per gram is the increase in plasma D:H from the dehydrated baseline (0 min) divided by the cumulative D_2_O ingested, during the 4 h rehydration period with either a sugar-sweetened oral rehydration solution (SS-ORS), sugar-free oral rehydration solution (SF-ORS), or water.

## Data Availability

The data presented in this study is available on request from the corresponding author. The data is not publicly available due to privacy restrictions.

## Supplementary Materials

The following supporting information can be downloaded at: https://www.mdpi.com/article/10.3390/nu18213601/s1, Figure S1: Absolute plasma D_2_O enrichment over the 4-h rehydration period with either a sugar-sweetened oral rehydration solution (SS-ORS), sugar-free oral rehydration solution (SF-ORS), or water; Table S1: Total amount of each ingredient consumed during the 4-h rehydration period; Table S2: Effect Sizes (Hedges’ g) for the Primary Outcomes; Table S3: Urine Electrolytes and Osmolality; Table S4: Perceptual rating.

## Abbreviations

The following abbreviations are used in this manuscript:

ASCT2	Sodium-dependent amino acid transporter 2
B^0^AT1	Sodium-dependent neutral amino acid transporter
BMI	Body mass index
D_2_O	Deuterium oxide
DEH	Post-dehydration baseline
EUH	Euhydration baseline
HSD	Honestly significant difference (Tukey’s HSD)
ORS	Oral rehydration solution
SD	Standard deviation
SE	Standard error
SF-ORS	Sugar-free oral rehydration solution
SGLT1	Sodium-glucose co-transporter 1
SS-ORS	Sugar-sweetened oral rehydration solution
USG	Urine specific gravity
VAS	Visual analog scale
VSMOW	Vienna Standard Mean Ocean Water
W	Water
