# Supplementary material for: Novel Allulose- and Amino Acid-Containing Sugar-Free Electrolyte Beverage Improves Body Water Restoration in Ad Libitum Rehydration Following Exercise-Induced Dehydration
Source: Nutrients. 2026 Oct 30;18(21):3601. doi: 10.3390/nu18213601 (PMC13765212; doi:10.3390/nu18213601)
Supplement: Supplementary file 1 [file nutrients-18-03601-s001.zip › nutrients-4591182-supplementary.pdf]

Figure S1. Absolute plasma D_2O enrichment over the 4-h rehydration period with either a sugar-sweetened oral rehydration solution (SS-ORS), sugar-free oral rehydration solution (SF-ORS), or water.

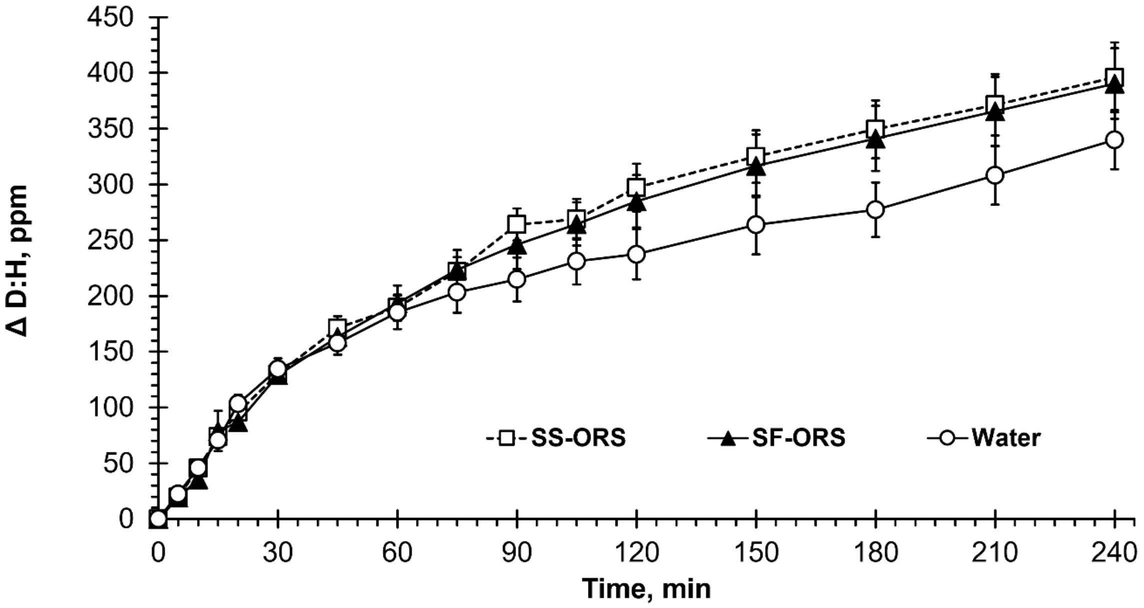

Data are presented as mean±SE (n = 20; 9 females). Plasma D_2O enrichment is the increase in plasma D:H from the dehydrated baseline (0 min), in ppm.

Table S1. Total amount of each ingredient consumed during the 4-h rehydration period.

Ingredient	SS-ORS	SF-ORS	Water
**Beverage consumed, mL**	1,918±713 (656–3,055)	1,826±728 (871–3,402)	1,603±567 (739–2,980)
**Carbohydrate, g**	49.9±18.5 (17.1–79.4)	—	—
**Allulose, g**	—	14.6±5.8 (7.0–27.2)	—
**L-glutamine + L-alanine, g**	—	14.6±5.8 (7.0–27.2)	—
**Sodium, mmol**	94.0±34.9 (32.1–149.7)	84.0±33.5 (40.1–156.5)	—
**Sodium, mg**	2,160±803 (739–3,441)	1,931±770 (921–3,598)	—
**Potassium, mmol**	36.4±13.6 (12.5–58.0)	34.7±13.8 (16.5–64.6)	—
**Potassium, mg**	1,425±530 (487–2,270)	1,356±541 (647–2,527)	—
**Energy, kJ**	802±298 (274–1,277)	305±122 (145–568)	—
**Energy, kcal**	192±71 (66–305)	73±29 (35–136)	—
**D_2O, g**	19.2±7.1 (6.6–30.6)	18.3±7.3 (8.7–34.0)	16.0±5.7 (7.4–29.8)

Data are presented as mean±SD (range) (n = 20; 9 females). Amounts were calculated for each participant as total beverage intake during the 4-h rehydration period (500-mL standardized bolus plus ad libitum intake) multiplied by the concentration of each ingredient (Table 1), for a sugar-sweetened oral rehydration solution (SS-ORS), sugar-free oral rehydration solution (SF-ORS), or water. Carbohydrate is glucose/sucrose; L-glutamine + L-alanine is the combined amino acid content (1:1 ratio).

Table S2. Effect Sizes (Hedges' *g*) for the Primary Outcomes.

Outcome	Time	SF-ORS vs. Water	SS-ORS vs. Water	SF-ORS vs. SS-ORS
**Total fluid intake**	240 min	0.33	0.47	−0.12
**Cumulative urine output**	180 min	−0.21	0.25	−0.43
	240 min	−0.39	0.05	−0.38
**Fluid retention**	180 min	0.57	0.40	0.21
	240 min	0.79	0.54	0.28
**Body weight change**	180 min	0.80	0.48	0.45
	240 min	0.90	0.47	0.53
	Over 4 h	0.76	0.53	0.34
**D_2O enrichment per g ingested**	Over 4 h	−0.21	−0.41	0.17

Hedges' *g* was calculated from the raw data (*n* = 20; 9 females) as the mean paired difference divided by the average SD of the two conditions with a small-sample correction. Positive values indicate a higher value for the first beverage in each comparison. Over 4 h, each participant's mean across the rehydration time points (30–240 min). Values of 0.2, 0.5, and 0.8 indicate small, medium, and large effects, respectively. SS-ORS, sugar-sweetened oral rehydration solution; SF-ORS, sugar-free oral rehydration solution.

Table S3. Urine Electrolytes and Osmolality

Time, min	EUH	DEH	60	120	180	240
**Urine Sodium, mmol/L**						
SS-ORS	38±26	36±37	55±35	45±43	44±35	62±42
SF-ORS	42±44	32±42	68±43	53±34	53±30	62±23
Water	45±33	34±39	55±48	27±28	29±22	26±17
**Sodium Excretion, mmol**						
SS-ORS			8±5	11±6	10±5	10±6
SF-ORS			8±4	12±6	11±6	12±6
Water			7±5	7±4	7±3	7±4
**Cumulative Sodium Excretion, mmol**						
SS-ORS			8±5	19±10	30±15	40±19*
SF-ORS			8±4	20±9	32±13	43±19*
Water			7±5	15±8	21±11	29±13
**Urine Potassium, mmol/L**						
SS-ORS	20±20	30±26	31±24	24±18	30±24	38±26
SF-ORS	21±26	22±18	37±28	33±27	31±21	34±15
Water	25±21	36±30	41±49	19±25	20±28	14±10
**Potassium Excretion, mmol**						
SS-ORS			4±2	6±3	7±4*	7±4
SF-ORS			4±2	7±4*	7±4*	7±4
Water			4±3	4±4	4±4	4±3
**Cumulative Potassium Excretion, mmol**						
SS-ORS			4±2	10±5	18±8	24±11*
SF-ORS			4±2	12±6	18±9	25±11*
Water			4±3	8±6	12±9	16±11
**Urine Chloride, mmol/L**						
SS-ORS	46±44	53±59	65±45	50±49	54±45	73±48
SF-ORS	47±58	42±53	81±56	65±46	66±38	74±29
Water	55±39	56±58	73±63	37±46	38±35	30±18
**Chloride Excretion, mmol**						
SS-ORS			9±5	11±5	12±5	12±6
SF-ORS			10±5	15±7*	14±7*	14±7*
Water			9±6	9±7	8±5	9±5
**Cumulative Chloride Excretion, mmol**						
SS-ORS			9±5	21±9	33±13	44±16
SF-ORS			10±5	25±10	39±15	52±21*

Time, min	EUH	DEH	60	120	180	240
**Water**			9±6	18±12	27±17	35±18
**Urine Osmolality, mmol/kg H_2O**						
**SS-ORS**	313±224	323±250	375±231	209±186	265±198	349±223
**SF-ORS**	277±235	264±211	430±223	306±178	319±161	378±148
**Water**	304±216	330±250	370±284	170±109	158±91	184±112
**Osmotic Excretion, mmol**						
**SS-ORS**			59±53	61±65	71±59	56±31
**SF-ORS**			54±19	71±26	68±26	69±32
**Water**			45±17	46±24	36±12	48±26
**Cumulative Osmotic Excretion, mmol**						
**SS-ORS**			59±53	120±92	191±143	247±168
**SF-ORS**			54±19	125±43	193±62	262±84*
**Water**			45±17	90±31	126±34	175±44

Data are presented as mean±SD (n = 20; 9 females). Urine electrolytes and osmolality are reported at euhydration baseline (EUH; before exercise in the heat), post-dehydration baseline (DEH), and throughout the 4-h rehydration period with either a sugar-sweetened oral rehydration solution (SS-ORS), sugar-free oral rehydration solution (SF-ORS), or water. Statistical significance was set at $P<0.05$. *, indicates the significant difference compared to water within the time point.

Table S4. Perceptual rating

Time, min	EUH	DEH	60	120	180	240
			Thirst, mm			
SS-ORS	41±32	60±36	32±24	39±27	36±25	28±18
SF-ORS	43±31	73±40	45±26	41±26	41±26	31±27
Water	30±22	71±32	44±25	46±25	43±28	25±20
			Stomach Fullness, mm			
SS-ORS	70±25	56±22	61±27	41±26	28±19	22±18
SF-ORS	62±21	52±25	43±31	45±29	28±26	21±20
Water	59±30	53±30	58±30	37±28	26±24	25±29
			Taste Pleasantness, mm			
SS-ORS		88±25	82±24	78±27	68±30	68±35
SF-ORS		88±30	82±30	79±35	74±34	69±34
Water		64±30	61±31	56±37	57±38	51±36

Data are presented as mean ± SD (n = 20; 9 females). Thirst, stomach fullness and taste pleasantness are reported at euhydration baseline (EUH; before exercise in the heat), post-dehydration baseline (DEH), and throughout the 4-h rehydration period with either a sugar-sweetened oral rehydration solution (SS-ORS), sugar-free oral rehydration solution (SF-ORS), or water. Statistical significance was set at $P<0.05$.
